# Retrospective study of nonmucinous appendiceal adenocarcinomas: role of systemic chemotherapy and cytoreductive surgery

**DOI:** 10.1186/s12885-017-3327-0

**Published:** 2017-05-15

**Authors:** Marc Uemura, Wei Qiao, Keith Fournier, Jeffrey Morris, Paul Mansfield, Cathy Eng, Richard E Royal, Robert A Wolff, Kanwal Raghav, Gary N Mann, Michael J Overman

**Affiliations:** 1 Department of Gastrointestinal Medical Oncology, The University of Texas MD Anderson Cancer Center, 1515 Holcombe Blvd., Unit 0426, Houston, TX 77030 USA; 2 Department of Biostatistics, The University of Texas MD Anderson Cancer Center, Houston, TX USA; 3 Department of Surgical Oncology, The University of Texas MD Anderson Cancer Center, Houston, TX USA

**Keywords:** Nonmucinous, Mucinous, Appendiceal, Eytoreductive surgery, Chemotherapy

## Abstract

**Background:**

Mucinous appendiceal adenocarcinomas (AAs) are the most common histological subset of AAs. Nonmucinous AAs have been infrequently studied. We performed a single-center retrospective study to investigate this histological subtype.

**Methods:**

We reviewed 172 patient records with nonmucinous AAs treated at MD Anderson Cancer Center from Jan, 1990 to Jun, 2015 and recorded patient demographics, tumor characteristics, treatment, and outcomes. Response rate (RR) was assessed semi-quantitatively (response/no response) according to the treating physician’s findings. Survival outcomes were calculated using the Kaplan-Meier product-limit method and compared using the log-rank test.

**Results:**

Median age at diagnosis was 52.9 years. Most patients presented with advanced-stage disease: stage I-II (35%), stage III (15%), and stage IV (50%). Moderate and poorly differentiated histology was seen in 56% and 44% tumors, respectively. Median overall survival (OS) of all patients was stage-dependent and was 88.5, 39.2, and 28.3 months for stages I-II, stage III, and stage IV disease, respectively (*p* < 0.0001). In patients with metastatic disease, only 10% had extraperitoneal disease without peritoneal involvement. Cytoreductive surgery (CRS) was attempted in 31/69 (45%) patients with disease confined to the peritoneum. Complete CRS was achieved in 18. Median OS for patients receiving complete CRS was 48.6 months. Systemic chemotherapy was administered to 109 (86%) patients with metastatic disease; a large majority of patients received either an oxaliplatin-based (55%) or irinotecan-based (27%) regimen. Chemotherapy resulted in a semi-quantitative RR of 54% and median time to progression (TTP) of 9.4 months (95% CI, 8.03–11.50). Patients who received combination chemotherapy (either oxaliplatin or irinotecan-based) showed significantly longer median OS (*p* = 0.003), compared to those receiving fluoropyrimidine monotherapy.

**Conclusions:**

This is one of the first studies to report specifically on nonmucinous AAs. Nonmucinous AAs presented with moderate or poorly differentiated histology with a predilection for peritoneal metastasis. Systemic chemotherapy is active in this AA subtype. Though CRS was infrequently used, complete CRS appears beneficial and warrants further investigation.

## Background

Appendiceal cancers are rare, representing 1% of intestinal tumors. They are usually discovered incidentally during appendectomy surgery for appendicitis [1]. Approximately two-thirds are adenocarcinomas, and are histologically classified into mucinous and nonmucinous subtypes. Recent observational studies of data from both the Surveillance, Epidemiology, and End Results (SEER) database and the National Cancer Data Base (NCDB) have shown similar overall survival (OS) and cancer-specific survival (unadjusted for grade and stage) for patients with mucinous and nonmucinous appendiceal tumors [2, 3]. However, when patients were stratified by histological grade and disease stage, marked differences in outcomes were noted. Particularly, the 5-year OS rate for patients with stage IV disease was significantly lower for those with nonmucinous (15%) than for those with mucinous tumors (41%). The differences were even more pronounced when well-differentiated stage IV appendiceal adenocarcinomas (AAs) were compared by histologic type: patients with nonmucinous well-differentiated stage IV disease had worse median OS (2.2 years) than those with mucinous (6.4 years) disease [3]. These findings suggest that nonmucinous and mucinous AAs may have differences in tumor biology, but observational data cannot evaluate the impact of treatment across these histological subtypes.

Although many studies have evaluated AAs, most derive from surgical literature and focus on surgical management and outcomes of mucinous, not nonmucinous AAs [4–6]. Because of this, there is a paucity of data describing the clinical features and treatment patterns of nonmucinous AAs. Though mucinous AA are known to have a predilection for peritoneal dissemination, the pattern of metastatic disease has not yet been described for nonmucinous AAs [7]. In patients who have mucinous AA with peritoneal dissemination (stage IV disease) cytoreductive surgery (CRS) and hyperthermic intraperitoneal chemotherapy (HIPEC) have been proposed as standard of care, especially for those with low-grade tumors [4, 8]. Whether this practice also holds true for nonmucinous AAs is unknown. Similarly, the efficacy of systemic chemotherapy in the treatment of AAs is uncertain, as the benefits appear to differ depending on histology (mucinous vs. nonmucinous) and tumor grade. A population-based study using the NCDB demonstrated no benefit from systemic chemotherapy for stage IV mucinous tumors but did show a benefit for the nonmucinous subtype (hazard ratio [HR], 0.73; 95% confidence interval [CI], 0.65–0.83; *p <* 0.0001) [3]. Though several other retrospective studies [9–12] have examined systemic chemotherapy in AA, none have described outcomes specifically in patients with nonmucinous AA.

Given the lack of published data on the nonmucinous AA subtype, we examined the natural history, outcomes, and treatment response for patients with these tumors. We sought to clinically describe this histological subtype and examine the treatment benefit of systemic chemotherapy and CRS on OS and time to progression (TTP).

## Methods

### Patient data collection

The Institutional Review Board of The University of Texas-MD Anderson Cancer Center (MDACC) approved this retrospective analysis with informed consent waiver. We reviewed MDACC tumor registry records to identify all patients diagnosed/treated with nonmucinous AA between Jan/1990 and June/2015. Included patients were ≥18 years of age at diagnosis. In all cases, the original appendiceal pathology specimen from either appendectomy or hemicolectomy procedures were obtained and reviewed; only cases with confirmed nonmucinous appendiceal histology were included. Signet ring cell carcinoma (i.e. signet ring cells >50% tumor) was excluded.

We extracted demographic and clinical characteristics of included patients from their medical records, including age, sex, ethnicity, tumor characteristics, treatment history, therapy response, disease progression, and survival status. We defined therapy response semi-quantitatively (response, no response, or stable disease) based upon radiographic changes in tumor size from radiology reports or clinician notes. In general patients underwent radiographic evaluation at approximately two-month intervals.

For patients with peritoneal metastases who underwent CRS, CRS was considered complete if there was <0.25 cm of visible residual disease after surgery. Patients with complete CRS were further stratified into 2 groups: those with a completeness of cytoreduction score (CC score) of 0 (no residual disease) and those with a score of CC-1 (< 0.25 cm of residual disease). Incomplete CRS was defined by the presence of CC-2 to CC-4 (≥ 0.25 cm of residual disease).

We subclassified patients with metastatic disease into 3 categories: those with peritoneal-only disease, those with extraperitoneal-only disease, and those with both peritoneal and extraperitoneal disease. Appropriate radiographic imaging or biopsy confirmed each metastatic disease site.

For inclusion in the systemic chemotherapy cohort, patients had to have visible disease on imaging studies, received at least one cycle of chemotherapy, and had to have a follow-up imaging evaluation after chemotherapy.

### Statistical analysis

TTP, relapse-free survival (RFS), and OS were calculated using the Kaplan-Meier product-limit method, and OS rates were compared using the log-rank test. For analysis of the entire study population, OS was measured from the date of diagnosis to the date of death. Patients lost to follow-up were censured. For the systemic chemotherapy cohort, TTP and OS were calculated from the date of first chemotherapy administration. For the CRS group, OS was calculated from the date of CRS to death or last follow-up. The chi-square test and Fisher’s exact test were used to assess relationships between categorical variables. The variables with potentially significant effect (*P* ≤ 0.1 or *P* ≤ 0.05) in univariate analysis were selected as candidates to put in the final multivariate models; variables with particular clinical interest were also considered in the final model. All *p* values were two-sided, and *p* values <0.05 were considered statistically significant. We used GraphPad Software Prism version 6 to create the Kaplan-Meier curves; all the statistical analyses were performed with SAS version 9.4.

## Results

We identified 172 patients with nonmucinous AA. Clinical characteristics are summarized in Table 1. The median age at diagnosis was 52.9 years (range: 26–82), with a 1:1 male-to-female ratio. Half of patients had stage IV disease at diagnosis. Fifty-six percent of patients had moderately differentiated tumors and 44% had poorly differentiated tumors.

**Table 1:** Demographic and clinical characteristics of 172 patients with nonmucinous appendiceal adenocarcinoma

Characteristic	Number of patients (%)
Median age at diagnosis	52.9 years (range: 26–82)
Sex	
Male	86/172 (50)
Female	86/172 (50)
Clinical stage at diagnosis	
I	3/172 (2)
II	57/172 (33)
III	26/172 (15)
IV	86/172 (50)
Histological grade	
Well differentiated	0/172 (0)
Moderately differentiated	96/172 (56)
Poorly differentiated	76/172 (44)
Race/ethnicity	
White	150/172 (87)
Hispanic	7/172 (4)
Black	12/172 (7)
Asian	3/172 (2)
Presence of signet ring cells	42/172 (24)
Presence of goblet cells	13/172 (8)
Presence of metastatic disease^a^	127/172 (74)
Sites of metastatic disease	
Peritoneal only	69/127 (54)
Extraperitoneal only	13/127 (10)
Peritoneal + extraperitoneal	45/127 (35)
CRS for metastatic disease	31/127 (24)
Complete (CC-0/1)	18/31 (58)
Incomplete (≥ CC-2)	13/31 (42)

^a^Metastatic disease categories include patients with stage IV disease at initial presentation and those with stage I-III disease who developed metastatic disease later in the clinical course.

Abbreviations: *CRS* cytoreductive surgery, *CC* completeness of cytoreduction

**Fig. 1:**
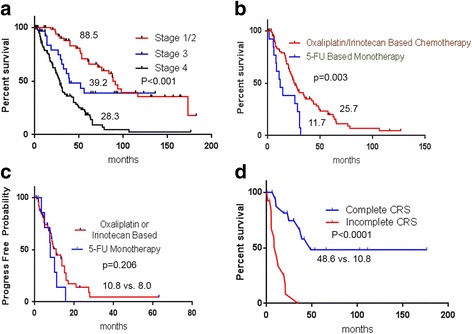
(**a**) Kaplan-Meier curve showing overall survival (months) in 172 patients with nonmucinous appendiceal adenocarcinoma by clinical stage at diagnosis. (Medians are indicated on curves). (**b**-**c**) Kaplan-Meier curves showing overall survival and time to progression for 109 patients with metastatic nonmucinous appendiceal adenocarcinoma by type of systemic chemotherapy (Medians are indicated on curves); 5-FU, 5-fluorouracil. (**d**) Kaplan-Meier curve showing overall survival for 31 patients who underwent complete or incomplete cytoreductive surgery (CRS)

The median time to last follow-up was 39.1 months (range: 0.8–182.5). The median OS for the entire study cohort was 46.7 months. The median OS duration stratified by disease stage at diagnosis was 88.5 months (95% CI, 61.4–174.3) for stages I-II, 39.2 months (95% CI, 29.1–not reached) for stage III, and 28.3 months (95% CI, 23.1–32.2) for stage IV (Figure 1a). The 3-year OS rate by stage was 87% for stages I-II, 55% for stage III, and 36% for stage IV disease.

Of the 127 patients who presented with or later developed metastatic disease, 54% had peritoneal-only metastases, 35% had both peritoneal and extraperitoneal metastases, and 10% had extraperitoneal-only metastases. Patients with extraperitoneal-only metastases commonly had liver and lung involvement.

In our multivariate analysis of the entire cohort, lymph node involvement and the presence of distant metastasis at presentation predicted for shorter OS (Table 2). Poorly differentiated histology predicted shorter OS in our univariate analysis but not the multivariate analysis.

**Table 2:** Univariate and multivariate analyses of overall survival for 172 patients with nonmucinous appendiceal adenocarcinoma

Variable	Univariate			Multivariate		
	HR	(95% CI)	*P* value	HR	(95% CI)	*P* value
Presence of signet ring cells	1.59	(1.06–2.39)	0.03	0.97	(0.60–1.56)	0.90
Poorly differentiated histology	3.03	(2.02–4.53)	<0.01	0.52	(0.24–1.12)	0.10
Lymph node involvement (N1 vs. N0)	2.37	(1.38–4.06)	<0.01	2.20	(1.23–3.92)	0.01
Lymph node metastasis (Nx vs. N0)	4.63	(2.86–7.49)	<0.01	3.12	(1.52–6.44)	<0.01
Advanced T stage (T4 vs. T1-T3)	1.64	(0.94–2.85)	0.08	1.70	(0.04–3.10)	0.08
Distant metastasis	3.58	(2.36–5.41)	<0.01	2.87	(1.28–6.44)	0.01
Age > or = 50 yrs. vs. < 50 yrs.	1.09	(0.73–1.61)	0.67			
Male vs. Female	1.41	(0.96–2.07)	0.08			
Race: White vs. Non-White	0.87	(0.49–1.55)	0.63			

Abbreviations: *HR* hazard ratio, *CI*, confidence interval

### Chemotherapy group

Systemic chemotherapy was administered to 109 (86%) of 127 patients who had stage IV disease at presentation or who later developed distant metastatic disease within the study period (Table 3). Of these, 60 (55%) patients initially received an oxaliplatin-based first-line regimen, 29 (27%) received an irinotecan-based first-line regimen, 13 (12%) received fluoropyrimidine alone, and 7 (6%) received “other” chemotherapy regimens. The semi-quantitative response rate for the 84 patients with measurable disease was 54%. Stable disease occurred in 19% and progressive disease in 26%. Though the analysis was strongly affected by selection bias, we found that the receipt of systemic chemotherapy was associated with a longer OS (HR, 6.61; 95% CI, 2.07–21.04; *p* < 0.01).

**Table 3:** First-line chemotherapy regimens for 109 patients with metastatic nonmucinous appendiceal adenocarcinoma

Regimen	Number of patients (%)
Oxaliplatin-based	60 (55)
FOLFOX or XELOX	18 (17)
FOLFOX or XELOX + bevacizumab	39 (36)
FOLFOX or XELOX + cetuximab	2 (2)
EOX	1 (1)
Irinotecan-based	29 (27)
FOLFIRI	5 (5)
Irinotecan alone	3 (3)
FOLFIRI + cetuximab	2 (2)
FOLFIRI + bevacizumab	12 (11)
FOLFIRI + bevacizumab + cetuximab	1 (1)
FOLFIRI + thalidomide	1 (1)
Irinotecan + cisplatin	4 (4)
Irinotecan + cetuximab	1 (1)
Fluoropyrimidine alone	13 (12)
Other	7 (6)
Irinotecan + oxaliplatin + bevacizumab	1 (1)
Carboplatin + paclitaxel	1 (1)
5-FU or capecitabine + cisplatin	4 (4)
5-FU + interferon	1 (1)

Abbreviations: *5-FU* 5-fluorouracil, *FOLFOX* 5-FU + oxaliplatin, *XELOX* capecitabine (Xeloda) + oxaliplatin, *EOX* epirubicin + oxaliplatin, *FOLFIRI* 5-FU + irinotecan

Patients who received an oxaliplatin- or irinotecan-based chemotherapy regimen had significantly longer median OS, but not TTP, than did patients who received fluoropyrimidine monotherapy. The median OS duration for patients who received oxaliplatin- or irinotecan-based chemotherapy regimens was 25.7 months, but only 11.7 months for those who received fluoropyrimidine monotherapy (*p* = 0.003) (Fig. 1b). The median TTP for the group that received oxaliplatin- or irinotecan-based chemotherapy was 10.8 months, while the median TTP for the fluoropyrimidine monotherapy group was 8.0 months (*p* = 0.206) (Fig. 1c). Notably, all patients in the oxaliplatin/irinotecan group received combination chemotherapy, except for 3 patients in the irinotecan group who received irinotecan monotherapy (Table 3).

The results of the univariate and multivariate analyses for the patient cohort that received systemic chemotherapy are presented in Table 4. The only variables that significantly predicted OS in our multivariate analysis were male sex (*p* = 0.03) and extraperitoneal-only metastasis (*p* = 0.03).

**Table 4:** Univariate and multivariate analyses of overall survival for 109 patients who received chemotherapy for metastatic appendiceal adenocarcinoma

Variable	Univariate			Multivariate		
	HR	(95% CI	*P* value	HR	(95% CI	*P*-value
Male gender	1.43	(0.93–2.21)	0.10	1.72	(1.05–2.84)	0.03
Presence of signet ring cells	1.19	(0.76–1.85)	0.27			
Lymph node metastasis	0.77	(0.46–1.29)	0.32			
Peritoneal only metastases	1.19	(0.77–1.85)	0.22	1.29	(0.76–2.19)	0.35
Extraperitoneal only metastases	2.07	(0.91–4.73)	0.08	2.64	(1.09–6.38)	0.03
Oxaliplatin or Irinotecan-based therapy vs. Fluoropyrimidine alone	2.55	(1.6–6.48)	0.05	2.08	(0.80–5.39)	0.13
Age > or = 50 yrs. vs. < 50 yrs.	0.99	(0.64–1.52)	0.95			
White vs. Non-White	0.71	(0.38–1.31)	0.27			
Poorly differentiated histology	1.01	(0.65–1.55)	0.97			
Cytoreductive surgery: Yes vs. No	0.74	(0.45–1.23)	0.25			

Abbreviations: *HR* hazard ratio, *CI* confidence interval

### CRS group

**Fig. 2:**
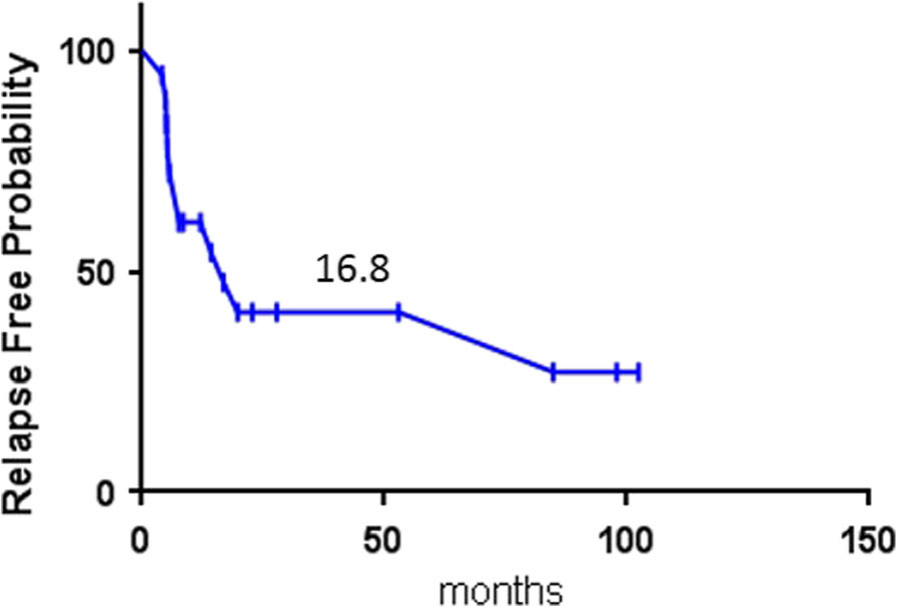
Kaplan-Meier curve showing relapse free survival for patients achieving complete CRS

Of the 127 patients with metastatic disease, 31 (24%) underwent CRS (48% moderate and 51% poor differentiation), and 18 had complete CRS. 12 of these patients who had complete CRS also HIPEC. Patients with complete CRS had a significantly longer median OS than did those with incomplete CRS (48.6 months vs. 10.8 months; *p* < 0.0001) (Fig. 1d). Perioperative systemic chemotherapy was administered to 27 of the 31 CRS patients. The most commonly used regimens among these patients included 5-fluorouracil plus oxaliplatin with or without bevacizumab (63%). The median time to last follow-up for surviving patients with complete CRS was 41.3 months, and the median relapse-free survival duration for patients who had complete CRS was 16.8 months (Fig. 2). At 1 and 3 years after complete CRS, 10 (56%) and 4 (22%) patients, respectively, remained free of disease.

## Discussion

Our single-center retrospective study is the first to evaluate the characteristics and treatment patterns of the nonmucinous AA subtype. Our results demonstrate that nonmucinous AAs tend to have high-grade histology and a strong predilection for peritoneal metastasis. These characteristics stand in contrast to those of mucinous AA, in which low-grade tumors predominate and to colorectal cancer, in which the liver is the most common metastatic site. Additionally, we show that systemic chemotherapy, particularly with oxaliplatin/irinotecan-based regimens has activity in nonmucinous AAs. Whether the low rate of attempted CRS (31/127 [24%] patients with metastatic disease) in our study reflects biological features of nonmucinous AA or treatment practice patterns cannot be determined from this study. Our data appears to support a beneficial impact from complete CRS. This combined with the high frequency of peritoneal-only metastasis in nonmucinous AA suggest that further study of CRS this this AA subtype is warranted.

The lack of data about the characteristics of nonmucinous AA has left clinicians to develop treatment approaches extrapolated from either colorectal cancer or mucinous AA. The effectiveness of these approaches in nonmucinous AA, however, is unknown. Our findings suggest that nonmucinous AA is biologically distinct from both mucinous AA and colorectal cancer. Notably, we found no well-differentiated nonmucinous AAs in our study. This is not surprising given that SEER and NCDB data demonstrate that the percentage of mucinous AAs with well-differentiated histology is nearly twice as high than that of nonmucinous AAs. Additionally, we found a predilection for peritoneal involvement, with 90% of patients having peritoneal metastatic disease. Mucinous AAs are usually characterized by slow growth and localized peritoneal spread that produces a clinical picture of pseudomyxoma peritonei; lymph node and extraperitoneal metastases are uncommon [11, 13]. In contrast, colorectal cancer most commonly metastasizes to the liver (up to 75% of all synchronous metastases), with peritoneal involvement in only a minority of cases [14–16].

Why mucinous and nonmucinous AAs display these clinical differences has not been elucidated. To date, only limited investigations into the molecular underpinnings of AAs have attempted to explain them. A 30-patient study at MDACC, which included 23 patients with mucinous and 7 with nonmucinous AA, demonstrated that the subtypes had similar molecular profiles, including a lack of microsatellite instability, p53 overexpression, and frequent *K-ras* mutations [7]. Another 149-patient study at MDACC showed that AAs have a distinct molecular makeup from colorectal cancer, with lower levels of microsatellite instability, higher cyclooxygenase-2 expression, and more frequent *K-ras* mutations [17]. Interestingly, the authors of that study also showed that the rates of *K-ras* mutations varied depending on the degree of histological differentiation. Clearly, more molecular analyses are needed to better understand the different clinical behavior and biology of these distinct AA subtypes.

We also demonstrated that systemic chemotherapy benefits some patients with nonmucinous AA, with a radiographic response observed in 54% of patients and a median TTP of 9.4 months. Combination chemotherapy with oxaliplatin or irinotecan appeared superior to fluoropyrimidine monotherapy, yielding a longer median OS and a trend toward improved TTP. Interestingly, the efficacy of systemic chemotherapy did not differ by histological grade; patients with moderately and poorly differentiated tumors had similar outcomes. Our observations of systemic chemotherapy activity in nonmucinous AA is consistent with a report based on NCDB data showing that systemic chemotherapy improved OS in patients with stage IV nonmucinous AA [3].

Lastly, CRS was attempted in only a minority of patients in our cohort. Whether this reflects biological applicability of this therapy to AA or the treatment pattern of utilizing CRS in mucinous AA is unknown. One randomized study demonstrated a longer median OS in patients with peritoneal carcinomatosis of colorectal cancer who underwent CRS [18]. The patients who received the greatest clinical benefit from CRS in our study were those with complete CRS (CC-0 or CC-1). These results are consistent with studies that have evaluated CRS in other intestinal tumors, where complete CRS predicted significant improvements in OS over incomplete CRS [4, 19]. Our data, along with the finding that there is a high rate of peritoneal-only disease in nonmucinous AA, suggest that increased use of CRS in nonmucinous AAs warrants additional study, with consideration given to early referral to a surgeon with specialization in peritoneal surface malignancies.

Overall, the results of our paper appear similar to treatment outcomes described for high grade mucinous AAs. Lieu, et al. previously described a 142-patient cohort of primarily poorly differentiated mucinous or signet ring cell AAs [9]. In this report, the overall response rate to systemic chemotherapy for patients with advanced disease was 44% using primarily fluoropyrimidine plus a platinum agent or irinotecan. In addition, a minority of patients with metastatic disease (21%) underwent a complete CRS. Similar to our findings a complete CRS was correlated with a significantly longer OS compared to those patients who did not achieve a complete CRS.

There are several limitations to this study. First, this analysis is retrospective, which can produce information and selection biases. Second, the rarity of nonmucinous AA represents a fundamental challenge with regard to the size of patient samples; the patient population we studied was relatively small, making definitive conclusions difficult. Third, this report is from a single academic center and may not be applicable to patients treated in other settings where differences in surgical expertise and availability of systemic therapies may affect treatment strategies. Finally, because of the long period studied, radiographic images for some patients were not available for review, and quantitative tumor response measures could not be determined.

## Conclusion

In conclusion, although nonmucinous AAs are difficult to study because they are rare, we have shown that nonmucinous AA has clinical features distinguishing it from both mucinous AA and colorectal cancer. At our institution, we treat nonmucinous AA using systemic chemotherapy regimens that are also used for colorectal cancer, and our retrospective data demonstrate a clinical benefit from this strategy. Nonetheless, the high rate of peritoneal-only dissemination and evidence of a benefit from complete CRS in nonmucinous AA suggest that further research on the effectiveness of CRS in treating nonmucinous AA is warranted.

## Key Message

Nonmucinous AAs have distinct clinicopathological features. Modern combination chemotherapy regimens that include oxaliplatin and irinotecan are efficatious. Outcomes after a complete CRS were encouraging and this treatment approach warrants further study in this histological subtype.

## Abbreviations

AA: Appendiceal adenocarcinoma
CC: Cytoreduction score
CI: Confidence interval
CRS: Cytoreductive surgery
HIPEC: Hyperthermic intraperitoneal chemotherapy
MDACC: MD Anderson Cancer Center
NCDB: National Cancer Data Base
OS: Overall survival
RFS: Relapse-free survival
RR: Response rate
SEER: Surveillance, epidemiology, and end results
TTP: Time to progression
